# Brain Metastases from Uterine Cervical and Endometrial Cancer

**DOI:** 10.3390/cancers13030519

**Published:** 2021-01-29

**Authors:** Mayumi Kobayashi Kato, Yasuhito Tanase, Masaya Uno, Mitsuya Ishikawa, Tomoyasu Kato

**Affiliations:** Department of Gynecology, National Cancer Center Hospital, 5-1-1 Tsukiji, Chuo-ku, Tokyo 104-0045, Japan; yatanase@ncc.go.jp (Y.T.); mauno@ncc.go.jp (M.U.); miishika@ncc.go.jp (M.I.); tokato@ncc.go.jp (T.K.)

**Keywords:** brain metastases, cervical cancer, endometrioid cancer, prognosis, treatment

## Abstract

**Simple Summary:**

This review investigated the prevalence, clinical characteristics, clinical presentation, diagnosis, treatment, and prognosis of patients with brain metastases from uterine cervical carcinoma (CC) and uterine endometrial carcinoma (EC). The findings of this review indicate the factors that can facilitate better treatment selection and, consequently, better outcomes in patients with CC and EC.

**Abstract:**

Reports on brain metastases (BMs) from uterine cervical carcinoma (CC) and uterine endometrial carcinoma (EC) have recently increased due to the development of massive databases and improvements in diagnostic procedures. This review separately investigates the prevalence, clinical characteristics, clinical presentation, diagnosis, treatment, and prognosis of BMs from CC and uterine endometrial carcinoma EC. For patients with CC, early-stage disease and poorly differentiated carcinoma lead to BMs, and elderly age, poor performance status, and multiple BMs are listed as poor prognostic factors. Advanced-stage disease and high-grade carcinoma are high-risk factors for BMs from EC, and multiple metastases and extracranial metastases, or unimodal therapies, are possibly factors indicating poor prognosis. There is no “most effective” therapy that has gained consensus for the treatment of BMs. Treatment decisions are based on clinical status, number of the metastases, tumor size, and metastases at distant organs. Surgical resection followed by adjuvant radiotherapy appears to be the best treatment approach to date. Stereotactic ablative radiation therapy has been increasingly associated with good outcomes in preserving cognitive functions. Despite treatment, patients died within 1 year after the BM diagnosis. BMs from uterine cancer remain quite rare, and the current evidence is limited; thus, further studies are needed.

## 1. Introduction

Malignancies arising from the uterus can be divided into two groups: uterine cervical carcinoma (CC) and uterine endometrial carcinoma (EC). The incidence of CC has gradually decreased because of the prevalence of the human papillomavirus vaccine and the screening of cervical cytology in almost all developed countries [1]. However, CC remains a leading cause of cancer death in women in developing countries, with approximately 570,000 cases and 311,000 deaths worldwide in 2018 [2]. Patients with early-stage CC and locally advanced CC have access to a standard treatment comprising a combination of surgery, radiotherapy, and chemotherapy; however, a standard treatment for patients with distant metastatic CC remains elusive. The 5-year survival rate for patients with distant metastatic CC is only 17.2%, compared to 91.5% for those with localized CC [3].

The rate of incidence of EC shows a significant upward trend relative to a declining birthrate and growing epidemic of obesity, especially in developed countries, with an estimated 382,000 new cases and 89,900 deaths reported worldwide in 2018 [2]. The tumor is limited to the uterine body in more than 70% cases and, therefore, surgical treatment has a good prognosis. When metastases do occur in EC, the treatment options are severely limited, and the 5-year survival rate of 16.3% for patients with distant metastases indicates an unfavorable prognosis [4]. Lymphatic spread is the main metastatic pathway of both CC and EC. CC metastasizes to para-aortic lymph nodes and supraclavicular lymph nodes more frequently than EC [5]. However, both of these cancers can spread hematogenously to distant organs, such as the lung, liver and bone. The previous studies showed that the frequency of metastases to these distant organs from CC is higher than that of metastases from EC [5,6].

Brain metastases (BMs) occur frequently with breast and lung cancers and malignant melanoma, which together account for more than 75% of all BMs [7,8]. In contrast, the brain is not a common site for both CC and EC metastases. Tumor cells from the genital tract spread via the hematogenous route: through the inferior vena cava, pulmonary artery, pulmonary veins, left atrium, left ventricle, and aorta to the brain [9]. Another possible route is from the veins of the pelvis to the paravertebral venous plexus, into the venous sinuses of the brain, and thereon to the brain parenchyma [7]. We conducted a search of the literature published over 50 years, between 1970 and 2020, to seek references to BMs from CC and EC. In a half-century, approximately 700 and 1100 cases of BMs from CC and EC, respectively, have been reported. The surveillance, epidemiology, and end results program (SEER) database undertook a search of metastasis information related to the liver, lung, bone, and brain since 2010, and found that the reported numbers of BMs from CC and EC have been increasing, and more than 85% of all cases were reported after 2010 in both CC and EC. This review summarizes reports from previous papers on BMs from CC and EC, with a focus on the prevalence, clinical characteristics, clinical presentation, diagnosis, treatment options, prognostic factors, and prognosis. 

## 2. Methods

This review was performed in accordance with the preferred reporting items for systematic reviews and meta-analyses (PRISMA) statement [10]. We searched the biomedical databases PubMed/Medline and the Cochrane library for all literature on BMs from CC and EC, published before June 2020. Terms used for the search included “brain metastases”, “gynecological cancers”, “cervical cancer”, “endometrial cancer,” and synonymous terms. Language was limited to English titles and abstracts. The search was restricted to 50 years between 1970 and 2020, to account for the rare occurrence of BMs in CC and EC. Data extraction was completed by the authors, and disagreements were solved by discussion.

### 2.1. Inclusion and Exclusion Criteria

All studies that provided detailed patient-specific information, such as clinical characteristics, clinical presentation, treatment, and prognosis of brain parenchyma metastases from CC and EC, were included. We also included database studies which described the number of patients and the incidence rates of brain parenchyma metastases from CC and EC. Publications presenting findings from autopsy and in vitro studies were excluded from this study. Studies reporting on leptomeningeal metastases from CC and EC were also excluded. 

### 2.2. Data Extraction

The following data were extracted from each study whenever possible: author and year of publication, number of patients in the study, clinicopathological features (age, histology of primary tumor, International Federation of Gynecology and Obstetrics (FIGO) stage, interval between the time of diagnosis of the primary tumor and BMs, presence of extracranial disease, number and site of BMs, symptoms, type of treatments (whole-brain radiotherapy (WBRT) alone, SRS alone, surgery alone, surgery plus WBRT, surgery plus SRS, and SRS plus WBRT), and prognosis). Data on clinicopathological features, treatment options, and prognosis of other gynecologic cancers, such as ovarian and vulvar cancers, were excluded, as were mixed data on CC and EC.

## 3. Results

The search strategy resulted in 329 articles on BMs form CC and 124 articles on BMs from EC on Pubmed. We did not find any publications on BMs from only CC or EC in the Cochrane Library, although 14 articles were found using the keyword “brain metastases.” Three articles on BMs from CC and one article on BMs from EC were excluded because they were only reviews, and did not report any new case. We also observed that only case reports, case series, and retrospective database studies provided information on number of patients, incidence rates, clinicopathological features, symptoms, and types of treatments. After screening the titles and abstracts, 83 full texts on BMs from CC and 78 full texts on BM from EC were selected and summarized (Table 1 and Table S1 [9,11,12,13,14,15,16,17,18,19,20,21,22,23,24,25,26,27,28,29,30,31,32,33,34,35,36,37,38,39,40,41,42,43,44,45,46,47,48,49,50,51,52,53,54,55,56,57,58,59,60,61,62,63,64,65,66,67,68,69,70,71,72,73,74,75,76,77,78,79,80,81,82,83,84,85,86], Table 2 and Table S2 [6,9,20,28,31,37,40,41,46,49,53,55,59,62,63,66,67,68,69,70,71,72,73,74,75,77,78,79,81,82,84,86,87,88,89,90,91,92,93,94,95,96,97,98,99,100,101,102,103,104,105,106,107,108,109,110,111,112,113,114,115,116,117,118,119,120,121,122,123,124,125,126,127,128,129,130,131]). Several case reports about the FIGO stage were lacking, and only histologic information was described. The rate of incidence of BMs in CC and EC within the overall incidence of CC and EC is estimated at 0.63% (range: 0.1–2.2%) and 0.7% (range: 0.2–1.2%), respectively (Table 1 and Table 2). The surveillance, epidemiology, and end results program (SEER) database undertook a search of metastasis information related to the liver, lung, bone, and brain from 2010 onwards, and found that the reported numbers of BMs from CC and EC have been increasing, and more than 85% of all cases were reported after 2010 in both CC and EC. However, the trend of the rate of incidence has not changed; the rate of incidence of BMs in CC ranged from 0.31% to 1.49% before 2010 (median: 0.76%), and was 0.10% to 2.2% after 2010 (median: 0.45%). Moreover, the rate of incidence of BMs in EC ranged from 0.30% to 1.16% before 2010 (median: 0.70%), and was 0.20% to 1.2% after 2010 (median: 0.70%). Despite improvements in diagnostic procedures and treatment options, such as surgery, radiotherapy, and chemotherapy, the prognosis remains poor in BMs, with a median overall survival of 5 and 7.5 months in CC and EC, respectively (Table 1 and Table 2).

We have summarized the prevalence and clinical characteristics, clinical presentation and diagnosis, treatment, prognostic factors, and prognosis of BMs from CC and EC in the following sections. Since there have been no meta-analyses or RCTs on the treatment protocols and prognostic factors of BMs from only gynecologic cancers, we have provided systematic reviews and RCTs on the treatment and prognostic factors of BMs in general. 

## 4. Cervical Cancer

### 4.1. Prevalence and Clinical Characteristics

Reports indicate that CC can metastasize hematogenously to distant organs, frequently including the lungs, liver, and bones [19,132,133]. BMs are rare and usually considered to be incurable. Generally, BMs comprise metastases to not only the brain parenchyma, but also to the leptomeninges [134]. Since 1970, 83 papers on the brain parenchymal metastases of CC have been published, including a total of 716 patients (Table 1 and Table S1). In contrast, there are less than 30 cases of leptomeningeal involvement reported in the English literature [36,81,135,136,137,138,139,140,141,142,143,144,145,146]. Autopsy studies have reported brain parenchymal metastases in 3–10% of CC patients [27,57,147], whereas the estimated frequency in the clinical setting ranged from 0.1% to 2.2% in this review; therefore, we mainly summarized information on brain parenchymal metastases.

The median age at the initial CC diagnosis was 48 years (range: 29–87 years). More than half of the patients had early-stage disease at the time of the diagnosis of BMs—25.5% and 33% had stage I and II, respectively, whereas most of the patients with EC had advanced disease [53]. The histologic grade was available for 38 patients [11,14,16,21,23,24,25,29,31,32,33,34,35,38,39,43,44,45,47,50,57,60,61,65], in whom 81.5% of BMs from CC have been reported to be poorly differentiated, whereas the remaining cases were to be well- or moderately differentiated. Histologic type was available in 224 patients [11,14,16,18,21,22,23,24,25,26,28,29,30,31,32,33,34,35,36,37,38,39,42,43,44,45,47,48,50,51,52,53,54,56,57,58,60,61,62,64,65,66,67,74,75,76,80,81,83], and squamous cell carcinoma (SCC) was the commonest cancer type in 62.9% of reported cases, followed by adenocarcinoma (AC; 19.6%) and adenosquamous carcinoma (ASC; 4.9%). These histologic types reflect the general population of the primary cervical lesion, whereas small cell neuroendocrine carcinoma (SCNEC) comprised 12.5% of all reported BMs from CC, which is significantly higher than rates in primary cervical lesions (2%) [65]. Among the various clinicopathological factors, highly invasive subtypes of CC may be the important factors for BMs due to abilities of tumor cells to proliferate.

The interval between the primary diagnosis and BMs was available for 216 patients [11,14,15,16,18,20,21,22,24,25,26,27,28,29,30,31,32,33,34,35,36,37,38,39,43,44,45,47,50,51,52,53,55,56,57,59,60,61,62,64,65,67,72,73,75,76,80,83], and ranged from −1 week to 386 months (median 24 months). A literature review published in 2012 reported that the median interval between the diagnosis of CC and BMs was 18 months [148]. An improvement in locoregional disease control led to a 6-month extension of the interval required for BMs to develop and become apparent [76]. In 94.4% (205/216) of patients, BMs were detected after the diagnosis of CC, although BMs were detected simultaneously with or prior to the diagnosis of CC in 11 patients [11,28,34,47,53,56,59,61,65,75]. 

### 4.2. Clinical Presentation and Diagnosis

The initial symptoms of BMs from CC may be nonspecific, and may include headaches (45.6%), syncope or seizures (14.0%), ataxia (11.8%), nausea/vomiting (11.0%), hemiparesis (10.3%), visual disturbance including diplopia (8.8%), generalized or extremity weakness (8.8%), altered mental status (6.6%), dizziness (5.1%), confusion (5.1%), speech impairment (5.1%), paresthesia, facial twitching, tinnitus, tremor and hemiballismus [11,14,18,20,21,23,24,25,26,28,29,31,32,33,34,35,37,38,39,42,43,44,45,47,50,51,52,55,56,57,58,60,61,63,64,65,76,80,81]. Intracranial edema resulted in papilledema due to the increased intracranial pressure, which is a common sign of a brain tumor [64,148]. These symptoms and signs are the same as those of other brain-occupying lesions.

Extracranial metastases were found in 340/528 (64.4%) patients, whereas 188/528 (35.6%) patients had no extracranial metastases [11,14,15,16,18,21,22,23,24,25,26,27,28,29,30,32,33,34,35,36,37,38,39,42,43,44,45,47,50,51,52,53,54,56,57,60,61,62,63,64,65,74,75,76,80,83,84]. More than half of the patients (143/246, 58.1%) had multiple lesions, whereas less than half (103/246 patients, 41.9%) had solitary lesions [11,14,16,18,20,21,22,23,24,25,26,27,28,29,30,31,32,33,34,35,36,37,38,39,42,43,44,45,46,47,50,51,52,53,54,55,56,57,58,59,60,61,62,64,65,72,73,74,75,76,80,81,83]. The proportion of patients without extracranial metastases had slightly decreased from 22% before 2010 to 19% after 2010. Moreover, the proportion of patients presenting with a single BM had decreased from 57.8% to 35.9% (before 2010 vs. after 2010). These changes are consistent with those of other solid tumors and are likely due to the increased use of brain MRI for clinical investigations [149,150].

Most of the BMs were located in the supratentorial region (cerebrum) in 121/165 (73.3%) patients, whereas the infratentorial region (cerebellum) was affected in 44/165 (26.7%) patients. Among the 79 patients for whom details of the supratentorial lesions were known, BMs were located in the parietal, frontal, occipital, and temporal lobes in 27 (34.2%), 21 (26.6%), 16 (20.3%), and 15 patients (19.0%), respectively [11,14,16,18,20,21,22,23,24,25,26,27,28,29,30,31,32,33,34,35,36,37,38,39,42,43,44,45,47,50,51,52,54,56,57,58,59,60,61,65,72,73,74,75,76,80,81]. The cerebellum, which occupies only 12.6% of total brain volume, contained a disproportionately high number of BMs for its size. This distribution shows the same tendency as BMs from pulmonary and gastrointestinal cancers. The rate of occurrence of BMs located in the parietal lobes is higher than that of BMs from other primary tumors [151]. Furthermore, when compared to other primaries, patients with CC were found to have a significantly higher percentage of leptomeningeal involvement [81].

Currently, routine brain imaging is not recommended in the guidelines published by the National Comprehensive Cancer Network or the American Society of Clinical Oncology for the surveillance of post-treatment CC patients, because of the very low incidence of BMs in patients with gynecological cancer [68,152]. Patients presenting with nonspecific symptoms, such as headache, may initially have symptoms that are misdiagnosed as the side effects of chemotherapy or other forms of treatment [153]. This increases the possibility of BMs from CC being underdiagnosed. When any one neurological symptom or sign is identified in CC patients, it is important to search immediately for BMs through brain imaging investigations.

### 4.3. Treatment 

For patients with BMs, there are several treatment options, such as whole-brain radiation therapy (WBRT), surgery, stereotactic radiosurgery (SRS), or a combination of these treatment options. In some cases, chemotherapy and immunotherapy are helpful, although there is no single most effective therapy that has received consensus for the treatment of patients with BMs from CC. Treatment depends on the number of the metastases, the clinical status, tumor size and metastases at distant organs.

#### 4.3.1. Whole-Brain Irradiation Therapy

In the 1980s, WBRT was suggested to reduce the tumor volume and treat micrometastases, which lead to the prevention of neurological death. Traditionally, WBRT alone was the most frequently used unimodal therapy. Patients with multiple BMs generally would be administered WBRT alone [148]. In a study comprising 12 patients with BMs from CC, 9 patients who received WBRT experienced symptomatic improvement; the median survival of patients who received WBRT was significantly longer than that of those who were treated with steroids alone (3.0 months vs. 0.5 months) [5,37]. Moreover, in the palliative setting, WBRT is a therapeutic option for patients with extensive systemic metastases, uncontrolled primary disease, or multifocal metastases. However, WBRT is associated with a lot of late complications, such as cerebral atrophy and leukoencephalopathy, and it is reported that 10–20% of patients who undergo WBRT develop cognitive dysfunction [154,155]. Prophylactic cranial irradiation (PCI) was recommended for patients with BMs from small cell neuroendocrine carcinoma (SCNEC) of CC after the 1980s, because the proportion of BMs was quite high, as described earlier [156,157]. However, among patients with small-cell lung cancer, the long-term neurocognitive effect of PCI, including a significant decline in memory (both immediate and delayed recall), have been reported [158,159]. Therefore, PCI might not be an important therapy even in SCNEC patients at present [160].

#### 4.3.2. Surgery

The best candidates for surgery seem to be those with a large solitary lesion measuring more than 3 cm, no evidence of extracranial disease or life-threatening metastases, or a need for histological diagnosis [21,38,161]. A disease-free interval of at least 12 months and a well-controlled primary tumor is desirable [26,28]. A literature review reports that most younger patients were treated with surgical resection [5,37]. 

#### 4.3.3. Stereotactic Radiosurgery

More recently, stereotactic radiosurgery (SRS) has become available [28,148], and this technique makes it possible to precisely deliver a high dose of gamma radiation to a small intracranial target without causing injury to the surrounding normal brain tissue. Thus, SRS, which is less invasive and less neurotoxic, may be used in inaccessible lesions and to control the symptoms of BMs [161,162]. Moreover, much published evidence of the long-term neurocognitive side effects of WBRT has led to the increasing use of SRS as the definitive therapy or in the postoperative setting [163,164,165]. In case reports or case series that have been published after 2000, SRS was included in the treatment of BMs from CC. Matsunaga et al. reported that the control rate and response rate at 6 months after SRS treatment were 96.4% and 93.0%, respectively [71]. Moreover, Chung et al. reported the use of SRS in the palliative setting for symptom relief and good quality of life. Thus, SRS may be a better option for palliative care than WBRT, which has a shorter administration duration than SRS [51,71].

#### 4.3.4. Chemotherapy

Chemotherapy has been used for the treatment of recurrent BMs, but has a limited influence on survival [166]. For patients with multiple BMs and other distant metastases, chemotherapy alone may be a first-line treatment because it may effectively control both types of metastases [52]. Cisplatin is the most common chemotherapy agent. However, topotecan could be a reasonable option because it has the ability to cross the blood–brain barrier [37,167]. No specific study has been conducted to identify the best chemotherapy regimen and dose for patients with BMs.

#### 4.3.5. Multimodal Therapy

In half a century, the treatment of BMs has changed from WBRT alone to multimodal therapy, including surgery followed by WBRT, surgery followed by SRS, SRS followed by WBRT, and chemotherapy after WBRT.

Traditionally, patients without extracranial metastases and single BMs >3 cm, especially young patients, would undergo surgical resection of the brain lesion followed by WBRT [34,37,38,39,52,62,168]. Surgery followed by WBRT prolongs both progression-free survival and overall survival [169,170,171] compared to surgery alone; therefore, surgery combined with WBRT was considered to be a first option of treatment for patients with BMs that need surgery. Recently, two phase III randomized controlled trials showed the noninferiority of surgery plus SRS to surgery plus WBRT for patients with one to four BMs, wherein a significant decline in cognitive function was seen more often with WBRT than with SRS, despite there being no difference in the overall survival in both groups [165,172]. Therefore, surgery followed by SRS can be selected as a new standard therapy for patients with one to four BMs.

Furthermore, SRS followed by WBRT appears to improve the survival time compared to SRS alone for BMs from CC. Chung et al. analyzed 13 patients—4 patients treated with SRS alone and 9 patients with SRS plus WBRT. The median survival from the diagnosis of BMs was 4.6 months (range: 1.0–15.9 months) for patients who were treated with SRS plus WBRT. On the other hand, for patients treated with SRS alone, the median survival was only 1.2 months. However, SRS alone was more preferred for patients with a relatively poor performance status, compared to SRS with WBRT [51]. Moreover, some case reports revealed similar results to those reported from the abovementioned studies [18,21,26,29,30,32,34,39,44,45,58]. However, considering the decline in learning and memory function, the findings from two randomized controlled trials led to recommendations of SRS monotherapy, especially among patients with one to three BMs [163,164]. The selection of surgery plus SRS and SRS ± WBRT must be decided individually for each case, with consideration of the size, location of the lesions, number, clinical condition, and availability of technology [34,68].

The median survival of patients who received chemotherapy after WBRT was significantly longer than in those who received no additional treatment after WBRT (4.4 vs. 0.9 months) in studies where the chemotherapy regimens included cisplatin, topotecan, etoposide, docetaxel, and cisplatin plus ifosfamide. The results of the studies suggest that chemotherapy after WBRT improves survival [5,37].

### 4.4. Prognostic Factors

Several validated scoring systems, including the graded prognostic assessment (GPA) and the recursive partitioning analysis (RPA), are used to estimate survival and guide treatment selection in other solid tumors [173,174]. The GPA and RPA scores are calculated and grouped for each patient according to historical prognostic factors. The GPA score includes the age, Karnofsky performance status (KPS), the number of metastases, and the presence of extracranial metastases. The RPA score includes the age, control of primary tumor, KPS and the presence of extracranial metastases. A retrospective study evaluated the GPA and RPA scores among patients with BMs from gynecologic malignancies. Only the KPS was associated with overall survival (*p* = 0.0002), whereas control of the primary tumor (*p* = 0.83) and the number of metastases (*p* = 0.51) were not significantly associated with survival time. For example, based on the GPA score, three of nine long-term survivors were placed into the worst prognostic group. Thus, these systems are not validated for the evaluation of patients with gynecologic cancer [63].

The favorable prognostic indicators in patients with BMs from CC seem to include age less than 50 years, good KPS, and single BM [34,52,83,175]. With regard to the presence of extracranial metastasis, conflicting findings have been reported. A retrospective cohort study conducted in 2016, including all gynecologic cancers, compared the survival between patients without and with extracranial metastases, and showed a median survival of 17 months versus 2 months after diagnosis of BMs in those without and with extracranial metastases, respectively [68]. On the other hand, a systematic review revealed that overall survival from the initial diagnosis of CC was significantly shorter for patients without extracranial metastases than for patients with extracranial lesions (7.63 months vs. 26.3 months, respectively; *p* = 0.0005); however, survival after diagnosis of BMs did not differ between the study groups. These findings suggest that, for patients with BMs from CC, the intracranial metastasis in itself represents a poor prognostic factor [176]. As the former study grouped together multiple gynecological cancers, it is speculated that the conflicting result was caused by the difference in the tumor biology of cervical tumors versus that of other gynecological tumors [176]. 

A novel scoring system, “Uterine GPA”, which can be applied for both CC and EC, was proposed in 2017 [75]. The Uterine GPA incorporates two simple clinical parameters: the numbers of BMs and extracranial metastases. Patients with more than five BMs and extracranial metastases had significantly poorer overall survival than patients with one to four BMs, or without extracranial metastases. However, as these data include patients with EC, it is unclear which prognostic factors would be appropriate exclusively for CC patients [75]. Prognostic factors of BMs from CC were summarized in Table 3.

### 4.5. Prognosis

The survival of BMs from CC is very poor. Of the 201 patients documented in this review, the median survival time after the diagnosis of BMs was 5 months (range: 0.5–120 months). There was no information on the overall survival of the patients in 20 case reports/case series. The survival times after the diagnosis of BMs with regard to the mode of therapy of BMs from CC were as follows [11,14,16,18,20,21,22,23,24,25,26,27,28,29,30,31,32,33,34,35,36,37,38,39,41,42,43,44,45,46,47,50,51,52,53,54,55,56,57,58,59,60,61,62,63,65,66,70,71,72,73,74,76,78,79,80,83,84]: no treatment, 0.25–3.3 months (median: 0.6 months; 5/13 patients); WBRT alone, 0.5–22 months (median: 4.5 months; 18/83 patients); SRS, either alone or combined with another treatment modality, 1–30 months (median: 6 months; 27/60 patients); and surgery followed by WBRT, 1–120 months (median, 7.5 months; 14/28 patients). A better survival outcome is achieved with surgery combined with WBRT. SRS either alone or in combination with WBRT showed greater benefits as described earlier in the Section 5.3 Treatment section [28,51,177,178]. Patients without any treatment had the worst survival outcome [148].

## 5. Endometrial Cancer

### 5.1. Prevalence and Clinical Characteristics

In EC, lymphatic spread usually occurs to the pelvic and para-aortic lymph nodes, and invades locally to the ovaries and surrounding tissues. Less often, EC spreads through the hematogenous route. The lungs, including the pleura and mediastinum, the liver, and bone are the most frequent sites of hematogenous metastases of EC [6,95].

Brain metastases from EC are rare, with only 115 cases reported in 35 published papers before 2012 [179]. However, the reported number of BMs from EC has been increasing in the recently published literature that used the SEER and National Cancer Database, with 1124 cases from 78 published papers from 1970 to date (Table 2 and Table S2 [6,9,28,31,37,40,41,46,49,53,55,59,62,63,66,67,68,69,70,71,72,73,74,75,77,78,79,81,82,84,86,87,88,89,90,91,92,93,94,95,96,97,98,99,100,101,102,103,104,105,106,107,108,109,110,111,112,113,114,115,116,117,118,119,120,121,122,123,124,125,126,127,128,129,130,131]). The rate of incidence of BMs in EC in the clinical setting is estimated at 0.7% (range: 0.2–1.2%) from this review, and is similar to the incidence rate of 1.1% that was reported from an autopsy study [180]. 

The median age at the initial EC diagnosis was 61 years (range: 32–84 years). Approximately 70% of the patients who developed BMs from EC had advanced-stage disease (stage III, 37.2% and stage IV, 31.2%) at the time of diagnosis. The histologic type was available in 391 patients [6,28,31,37,53,59,62,66,67,74,75,81,87,88,90,92,93,94,95,96,97,98,99,100,101,102,103,104,106,107,108,109,110,112,113,114,115,117,118,119,120,121,122,123,124,125,126,127,128,131], of whom 319 (81.5%) had adenocarcinomas and 74 (18.9%) had unfavorable cancer types, such as carcinosarcoma, leiomyosarcoma, small-cell carcinoma, and undifferentiated carcinoma. BM was more likely to occur in patients with carcinosarcoma and undifferentiated histological type than in the general population of patients with primary endometrial lesion [6]. Among the 192 patients for whom the histological grade was known, 74.5% had high-grade (Grade 3) disease [6,20,31,37,81,90,93,94,95,96,98,100,101,102,104,105,106,107,108,109,110,112,113,114,117,118,121,122,123,124,125,126,128,131], including 27 cases of serous adenocarcinoma and 5 cases of clear cell adenocarcinoma.

Data on the interval between the diagnosis of EC and BMs were available for 289 patients. The BM was identified after the diagnosis of EC with a medical interval of 22 months (range: 1.5–216 months) in 273 of the 289 patients (94.5%). In 6 patients, the primary and metastatic lesions were diagnosed simultaneously [72,93,96,108,112,122], and in 10 patients the brain metastases were detected before the primary lesion [28,31,96,100,101,106,118]. A retrospective study compared the risk of BMs between EC patients with or without lung or liver metastases, and showed that the presence of lung metastases and liver metastases conferred a significantly higher risk of BMs (4.9% vs. 0.1%, 3.9% vs. 0.1%, respectively) [6].

### 5.2. Clinical Presentation and Diagnosis

The symptoms of BMs from EC are the same as the symptoms and signs of other space-occupying brain lesions, including BMs from CC. In this review, the common presenting symptoms included headache (27.4%), weakness (22.1%), syncope/seizures (12.4%), visual disturbance including diplopia (9.7%), ataxia (8.8%), speech impairment (7.1%), dizziness (7.1%), altered mental status (7.1%), confusion (6.2%), hemiparesis (6.2%), nausea/vomiting, numbness, dysarthria, hyponatremia, stroke, and memory loss.

The type of BMs with regard to the presence of extracranial metastases was available for 770 patients [20,28,31,37,49,53,62,63,74,75,84,87,88,89,90,91,92,93,94,95,96,97,98,99,100,101,102,103,104,105,106,107,108,109,110,111,112,113,114,115,117,118,119,120,121,122,123,124,125,126,127,128,131], and extracranial metastases were seen in 346/770 (45.0%) of patients, whereas 424/770 (55.0%) patients had no extracranial metastases. Information on whether the metastasis was a single or multiple BMs was available for 411 patients [28,31,37,49,53,62,63,74,75,87,88,90,92,93,94,95,96,97,98,99,100,101,102,103,104,105,106,107,108,109,110,112,113,114,115,117,118,119,120,121,122,123,124,125,126,127,128,131]; more than half of the patients (224/411 patients, 54.5%) had multiple lesions, whereas less than half (187/411 patients, 45.5%) had solitary lesions. Moreover, whereas the proportion of patients without known extracranial metastases was 49.5% before 2010, this proportion decreased to 23.2% after 2010. Around 2010, a substantial decrease in the number of patients with a single BM was observed (68.0% vs. 42.1%), and this was observed for other solid tumors, as described earlier in the CC section. Data on the site of BMs, that is, whether the metastasis was supratentorial (cerebrum) or infratentorial (cerebellum) or both, were available for 195 patients [20,28,31,49,55,72,75,87,88,90,92,93,94,95,96,97,98,99,100,101,102,104,105,106,107,108,109,110,112,113,114,117,118,119,120,121,122,124,125,126,127,128,131]. Most of the BMs were located in the supratentorial region (cerebrum; 143/195 (73.3%) patients); in 50/195 (25.6%) and 12/195 (6.2%) patients, the BMs were in the infratentorial region (cerebellum) and in both sites, respectively. Among the 121 patients for whom details of the lesions in the supratentorial region were known, the BMs were located in the frontal, parietal, occipital, and temporal lobes in 47 patients (38.9%), 46 patients (38.0%), 30 patients (24.8%), and 15 patients (12.4%), respectively. BMs from EC favored the infratentorial area, similarly to the BMs from CC. Only three papers reported a total of four patients with leptomeningeal metastases from EC [37,81,135].

### 5.3. Treatment

The treatment modalities of BMs from EC included WBRT alone, surgery alone, surgery followed by WBRT, surgery followed by SRS, SRS alone, WBRT followed by SRS, and palliative care. The treatment selection should be based on performance status, localization of BMs, number of the BMs, and the presence of extracranial disease [62,179].

In studies of BMs from EC published in the literature, treatment approaches and modalities for BMs changed over prolonged periods of time. Traditionally, surgery followed by WBRT was used for cases with good performance status, solitary lesions >3 cm and well-controlled extracranial disease [181]. Patients with multiple (>5) BMs, unresectable tumors, or resection cavities received WBRT alone. 

In a series of 12 patients with BMs from EC, the BMs were treated by surgery followed by WBRT in four patients, WBRT in six patients, and no treatment in two patients. The authors observed that four patients who underwent surgery combined with WBRT had a longer survival (median: 8 months) than the six patients who had unimodal therapy with WBRT alone (median: 3 months) and the two patients who received no treatment (median: 2 months) [131]. A literature review conducted in 2016 reported the superiority of surgery followed by WBRT compared to unimodal treatment, based on the median survival of patients with a single BM and no extracerebral involvement (27 months vs. 3.5 months, *p* < 0.001) [127]. Another review of patients without extracranial lesions revealed that the 2-year survival rate after diagnosis of BMs was 77% in patients who had surgery plus radiotherapy, whereas it was 19% in the surgery alone and 20% in the WBRT alone groups (*p* = 0.003 and 0.001, respectively) [128]. Recently, as two phase III randomized controlled trials [165,172] showed the noninferiority of surgery plus SRS to surgery plus WBRT for patients with one to four BMs, which are both described in the CC section, future studies regarding the effectiveness of surgery plus SRS for patients with BM from EC are warranted.

Since 2001, SRS has been included in the treatment of BMs from EC in case series and singular case reports in the literature. SRS is a good treatment option for BMs that are generally 3 cm or smaller in diameter, spherical, and minimally invasive, especially in patients with isolated and single BMs who are unable to tolerate surgery or have surgically inaccessible deep lesions [84,114,116]. In addition, SRS provides good local control with less morbidity because of the reduced dose delivery to the surrounding brain tissue [108]. SRS treatment in two patients with BMs from EC was first reported in 2001. One patient died of the disease 15 months after the diagnosis of BM. The other patient was still alive without evidence of disease 171 months after she had received SRS [106]. A literature review reported that SRS was a part of the therapy for patients without extracranial metastases from EC. Two of the four patients underwent SRS, wherein one underwent surgical resection prior to SRS and the other did not. The survival times of these two patients were 15 and 29 months, respectively, which were indicative of relatively good prognoses [128]. Moreover, we found that SRS leads to similar favorable results [20,37,62,108,116].

Another meta-analysis showed that, in patients aged 50 years or younger, SRS alone is more beneficial than the combination of SRS with WBRT [182]. On the other hand, SRS plus WBRT is advantageous for patients older than 50 years because the recurrence rate is reduced. EC usually occurs in postmenopausal patients; therefore, such patients are often suitable candidates for SRS with WBRT. However, as we mentioned in the CC section, two randomized controlled trials recommended SRS alone for patients with one to three BMs due to the side effects of WBRT [163,164]. In the absence of prospective studies on EC, we suggest that aggressive multimodal treatment using SRS with WBRT should be considered for patients older than 50 years who have more than four BMs.

### 5.4. Prognostic Factors

Much of the literature reported on factors associated with survival after the diagnosis of BMs from EC. With regard to the type and the number of metastases, the survival times of patients without extracranial metastases were significantly longer than those of patients with extracranial metastases in several reports [20,28,37,110,179]. Moreover, survival time after diagnosis of single BMs was significantly longer than that with the diagnosis of multiple BMs in several reports [20,28,179]. The reason for this finding is not completely clear, but it may be because there is a bias toward the more aggressive treatment of patients without extracranial metastases and a single lesion [116]. On the other hand, two retrospective reviews reported no significant difference in survival as determined by either the type or the number of BMs [37,62]. A literature review summarized only the topic of BMs without extracranial lesions from EC [128]. The results showed that age, grade, tumor type, diagnosis time, disease-free interval, localization, and number of BMs were not predictive of the survival time after diagnosis of BMs. However, survival in the presence of a single lesion was better than that in the presence of multiple lesions among patients without extracranial metastases, and this survival advantage showed a statistical trend toward significance (*p* = 0.076).

In 2016, Uccella et al. pointed out that previous literature reviews had combined the cases of primary cerebral dissemination and secondary recurrence. They conducted analyses of possible prognostic factors associated with survival after diagnosis of BMs, which definitely distinguished between BMs as the primary metastases and secondary relapses, which is the subsequent site of recurrence. In the univariate analysis, the better prognostic factors were identified as follows: early-stage disease, no extracranial lesions, presence of single metastases, and treatment combining surgery and WBRT. Multivariate analyses revealed that only treatments combining surgery and WBRT were significantly associated with better survival (*p* = 0.001) [127]. However, all women in their review who were treated with the combination of surgery plus WBRT had a single brain lesion and no extracranial lesions. Therefore, they concluded that it is difficult to determine whether it was the disease characteristics or the treatment type that was responsible for the improved patient prognosis. Prognostic factors of the BMs from EC were summarized in Table 4.

### 5.5. Prognosis

The survival of BMs from EC is very poor, and depends on the status of the primary lesion, the presence of extracranial metastases, and the volume, number, and site of metastases in the brain parenchyma. The survival time after diagnosis of BMs from EC was available for 946 patients in the literature and ranged from 0.1 to 171 months (median: 7.5 months) [6,9,28,31,37,40,41,46,49,53,55,59,62,63,66,67,68,69,70,71,72,73,74,75,77,78,79,81,82,84,86,87,88,89,90,91,92,93,94,95,96,97,98,99,100,101,102,103,104,105,106,107,108,109,110,111,112,113,114,115,116,117,118,119,120,121,122,123,124,125,126,127,128,129,130,131].

The overall survival times after diagnosis of BMs according to the mode of therapy of BMs from EC documented in the literature were as follows: no treatment, 0–7.6 months (median: 1 month; 21/35 patients); surgery alone, 0.75–18 months (median: 3 months; 13/25 patients); WBRT alone, 0.25–30 months (median: 5 months; 60/89 patients); SRS, either alone or combined with another modality, 1–171 months (median: 9.75 months; 25/73 patients), and surgery followed by WBRT, 2.5–118 months (median: 15.5 months; 44/56 patients). Patients treated with multimodal therapy, including surgery or SRS plus WBRT, achieved relatively longer survival compared with those treated with WBRT alone or surgery alone.

## 6. Treatment Strategies and Future Perspectives for BMs from CC and EC

**Figure 1:**
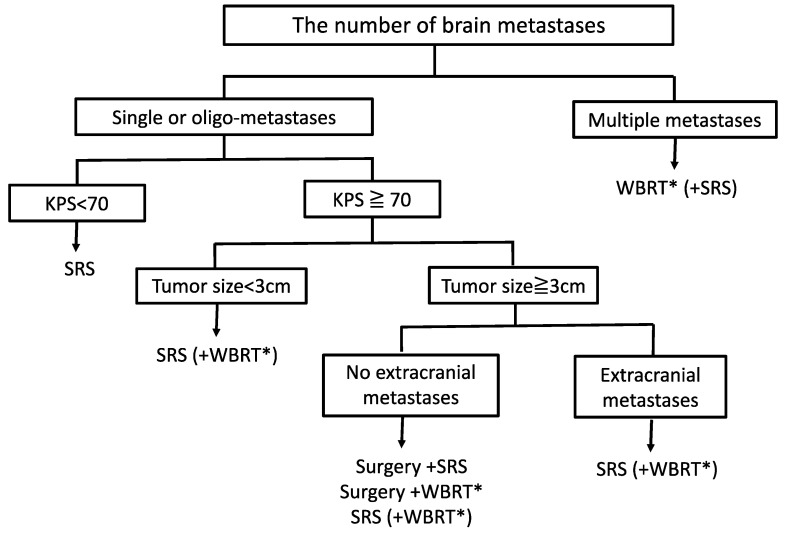
Treatment strategies for Brain metastases from cervical cancer and endometrial cancer. HA-WBRT: hippocampal avoidance using intensity-modulated radiotherapy during whole-brain radiotherapy, KPS: Karnofsky performance status, SRS: stereotactic radiosurgery, WBRT: whole-brain radiotherapy.

We have reviewed treatment options for patients with BMs from CC and EC, respectively. However, the treatment strategies for BMs from CC and EC are currently similar due to their rarity and the lack of meta-analysis or RCTs. We have provided our recommendations for treatment strategies of BMs from CC and EC in Figure 1, based on some treatment options that have been established for patients with BMs in general, not only for patients with BMs from CC and EC. Although only a few patients with CC and EC were included, there are several trials that show the effectiveness of SRS for single or oligo-metastases, as mentioned above [163,164,165,172]. On the other hand, no clinical trials have been found regarding the effectiveness of SRS alone for multiple metastases. Thus, we suggest that WBRT could be the best option to date. Recently, a phase III trial showed that hippocampal avoidance using intensity-modulated radiotherapy during whole-brain radiotherapy (HA-WBRT) better preserved cognitive function, and the patient reported less adverse events, without significantly affecting the progression free survival (PFS) and overall survival (OS), compared to WBRT. Although the trial did not include patients with BMs from CC and EC, HA-WBRT may be considered as the standard of care in patients with good performance status and no metastases invading the hippocampal region [183].

Recent studies on the treatment options for BMs take into account the adverse events following WBRT, and put a high value on the patient’s quality of life rather than PFS and OS. SRS or HA-WBRT prevent neurocognitive deterioration, providing a relatively acceptable quality of life in patients with BM compared with conventional WBRT. Phase III trials comparing SRS with HA-WBRT plus neurocognitive protective agents are ongoing (ClinicalTrials.gov. identifier: NCT03550391). Future trials on the effectiveness and safety of SRS or HA-WBRT should include a more homogenous cohort of patients with BM, such as those experiencing BM from CC and EC.

Although CC and EC are completely distinguishable cancers, treatment strategies for BMs from CC and EC are currently similar, as mentioned above. The difference between CC and EC is the regimen of systemic therapy. Thus, new approaches to systemic therapy may lead to separate research and treatment options in the fields of CC and EC. However, chemotherapy is not a treatment of first choice in BMs, since most drugs have difficulty crossing the blood–brain barrier (BBB) and reaching adequate therapeutic concentrations within the central nervous system. 

Molecular-targeted agents are exceptions to this generality, and there is diagnostic evidence of drug sensitivity. The vascular endothelial growth factor (VEGF) receptor inhibitor, bevacizumab, has limited activity as a single agent; however, it improves the delivery and potentiates the effects of other agents [184]. Several phase II clinical trials are ongoing to test the clinical benefit of combining bevacizumab with other agents for patients with BMs [185]. The use of the combination of bevacizumab with paclitaxel and cisplatin is expected for patients with BMs from CC in the near future. On the other hand, patients with BMs from EC have the potential to benefit significantly from nanomedicines. Efficient encapsulation of the drugs through nanomedicine enables drugs to cross the BBB [185]. At least 50 nanocarriers have been clinically approved, including Doxorubicin Hydrochloride Liposome [186]. Future studies on the effectiveness of these nanocarriers for BMs from EC are needed.

## 7. Limitation

The major limitation of this review was the lack of high-quality evidence, such as meta-analysis and RCTs, which prevented us from establishing definitive prognostic factors and treatment strategies for BMs from CC and EC. Additionally, considerable prior studies did not provide reports on the outcomes of treatments for CC and EC separately. This is because treatment options are surgically focused and, to date, studies regarding systemic therapy are not available for both CC and EC. These current situations make it difficult to conduct statistical analysis in our review. New strategies using molecular-targeted agents and nanomedicines are expected to separate research and treatment options in the field of CC and EC, respectively. However, our review conducted a systematic search and summarized all studies regarding BMs from CC and EC. This study can facilitate a better understanding of the prevalence, clinical characteristics, and presentation of uterine cancers, and also leads to better treatment selection and outcomes for patients with CC and EC.

## 8. Conclusions

We summarized BMs from CC and EC based on the aspects of prevalence, clinical characteristics, clinical presentation, diagnosis, treatment, prognostic factors, and prognosis. BMs from CC and EC remain a rare event, and the incidence rate has shown no substantial change over a half-century. With regard to prognostic factors, there are conflicting reports in both BMs from CC and those from EC. Younger age, good KPS, and single metastases seem to be favorable prognostic factors for patients with BMs from CC, whereas patients who had single metastases and no extracranial metastases, or surgery with WBRT, are candidates for good prognosis in BMs from EC.

A combination of surgery and WBRT, if feasible, seems to be the best option to improve survival rates in BMs from both CC and EC. Recently, it seems that SRS has gained an advantage over other therapeutic approaches in terms of both high survival rates and fewer cognitive and constitutional side effects. HA-WBRT is also another new treatment strategy to preserve cognitive function in patients with BM. Despite the availability of all treatment options, the median survival time from the diagnosis of BMs to death remains short. Thus far, there are no targeted therapies or nanomedicines for BMs from CC and EC. Future studies through large prospective randomized trials are expected to identify more effective treatment options. Moreover, identifying tumor-specific biomarkers of BMs from CC and EC will make it possible to prevent the occurrence of BMs.

## Figures and Tables

**Table 1:** Summary of clinicopathological features, treatments, and outcome data of patients with brain parenchyma metastases from cervical cancers documented in the literature.

	N		N
Median age at the initial Cx diagnosis (y)	48 (29–87)	Symptoms (%) (*n* = 136)	
		Headaches	62(45.6)
Incidence (%)	0.63 (0.1–2.2)	Syncope/seizures	19 (14.0)
		Ataxia	16 (11.8)
Histology (%) (*n* = 224)		Nausea/vomiting	15 (10.3)
SCC	141 (62.9)	Visual disturbance	12 (8.8)
AC	44 (19.6)	Weakness	12 (8.8)
ASC	11 (4.9)	Altered mental status	9 (6.6)
SCNEC	28 (12.5)	Dizziness	7 (5.1)
		Confusion	7 (5.1)
FIGO stage at diagnosis of BMs (%) (*n* = 208)		Speech impairment	7 (5.1)
I	53 (25.5)	Paresthesia	2
II	69 (33.0)	Facial twitching	2
III	41 (19.7)	Tinnitus	1
IV	45 (21.6)	Tremor	1
		Hemiballismus	1
Interval Cx to BM (m)	24 (−0.25–386)		
		Treatments	
Other metastases (%) (*n* = 528)		WBRT alone	83
No	188 (35.6)	SRS alone	49
Yes	340 (64.3)	Surgery alone	17
		Surgery plus WBRT	28
Single BM or Multiple BMs (%) (*n* = 246)		Surgery plus SRS	2
Single	103 (41.8)	SRS plus WBRT	9
Multiple	143 (58.1)	BSC	13
Site of BM (%) (*n* = 165)		Median survival (mo)	5 (0.5–120)
cerebrum	121(73.3)		
parietal lobe	27 (16.4)		
frontal lobe	21 (12.7)		
occipital lobe	16 (9.7)		
temporal lobe	15 (9.1)		
unknown	42		
cerebellum	44(26.7)		

AC: adenocarcinoma, ASC: adenosquamous carcinoma, BMs: brain metastases, BSC: best supportive care; Cx: cervical cancer, SCC: squamous cell carcinoma, SCNEC: small cell neuroendocrine carcinoma, SRS: stereotactic radiosurgery, WBRT: whole-brain radiotherapy.

**Table 2:** Summary of clinicopathological features, treatments, and outcome data of patients with brain parenchymal metastases from endometrial cancers documented in the literature.

	N		N
Median age at the initial EC diagnosis (y)	61 (32–84)	Site of BM (%) (*n* = 195)	
		cerebrum	143 (68.2)
Incidence (%)	0.7 (0.2–1.2)	frontal lobe	47 (24.1)
		parietal lobe	46 (23.6)
Histology (%) (*n* = 391)		occipital lobe	30 (15.4)
AC	319 (81.6)	temporal lobe	15 (7.7)
ASC	15 (3.8)	unknown	22
SCC	2 (0.6)	cerebellum	50 (25.6)
UD	7 (1.8)		
SCNEC	3 (0.8)	Symptoms (%) (*n* = 113)	
CS	31 (8.0)	Headaches	31 (27.4)
LS	8 (2.0)	Weakness	25 (22.1)
AS	2 (0.5)	Syncope/seizures	14 (12.4)
Sarcoma	4 (1.0)	Visual disturbance	11 (9.7)
		Ataxia	10 (8.8)
Histological grade (%) (*n* = 192)		Altered mental status	8 (7.1)
Grade1	17 (8.9)	Dizziness	8 (7.1)
Grade2	32 (16.7)	Speech impairment	8 (7.1)
Grade3	143 (74.5)	Confusion	7 (6.2)
		Hemiparesis	7 (6.2)
FIGO stage at diagnosis of BMs (%) (*n* = 253)		Nausea/vomiting	3
I	57 (22.5)	Numbness	3
II	23 (9.1)	Dysarthria	2
III	94 (37.2)	Hyponatremia	2
IV	79 (31.2)	Strokes	2
		Memory loss	1
Interval EC to BM (m)	18 (−3–216)		
		Treatments	
Other metastases (%) (*n* = 770)		WBRT alone	89
No	424 (55.0)	SRS alone	66
Yes	346 (45.0)	Surgery alone	25
		Surgery plus WBRT	56
Single BM or Multiple BMs (%) (*n* = 411)		Surgery plus SRS	2
Single	187 (45.5)	SRS plus WBRT	5
Multiple	224 (54.5)	BSC	35
		Median survival (mo)	7.5 (0.1–171)

AC: adenocarcinoma, AS: adenosarcoma, ASC: adenosquamous carcinoma, BMs: brain metastases, BSC: best supportive care, CS: carcinosarcoma, EC: endometrial cancer, LS: leiomyosarcoma, SCC: squamous cell carcinoma, SCNEC: small-cell neuroendocrine carcinoma, SRS: stereotactic radiosurgery, Surg: surgery, UD: undifferentiated carcinoma, WBRT: whole-brain radiotherapy.

**Table 3:** Prognostic factors of BMs from cervical cancers.

Favorable/Poor/Unknown	Prognostic Factors
Favorable	Age < 50 or Karnofsky performance status ≧ 70 or single BMs multimodal therapy
Poor	Multiple BMs and extracranial metastases
Unknown	The presence of extracranial metastases

**Table 4:** Prognostic factors of BMs from endometrial cancers.

Favorable/Poor/ Unknown	Prognostic Factors
Favorable	Single BMs and no extracranial metastases, multimodal therapy
Poor	Multiple BMs and extracranial metastases
Unknown	Stage, the number of metastases, the presence of extracranial metastases

## Data Availability

The data presented in this study are available in Tables S1 and S2.

## Supplementary Materials

The following are available online at https://www.mdpi.com/2072-6694/13/3/519/s1, Table S1: Clinicopathological features, treatments and outcome data of patients with brain parenchyma metastases from cervical carcinoma documented in the literature, Table S2: Clinicopathological features, treatments and outcome data of patients with brain parenchyma metastases from endometrial carcinoma documented in the literature.
