# Supplementary material for: Brain Metastases from Uterine Cervical and Endometrial Cancer
Source: Cancers (Basel). 2021 Jan 29;13(3):519. doi: 10.3390/cancers13030519 (PMC7866278; doi:10.3390/cancers13030519)
Supplement: Supplementary file 1 [file cancers-13-00519-s001.pdf]

Supplementary table1. Clinicopathological features, treatments and outcome data of patients with brain parenchyma metastases from cervical carcinoma documented in the literature.

Author	BMs(N)	Incidence	Histotype (N)	FIGO stage (N)	Interval Cx to BM (mo)	Other metastases	Median age (yrs)	Single BM/ Multiple BMs (N)	Most common symptoms	Site of BM	Treatments	Median survival (months)
Buchsbaum 1972	1		SCC (poorly)	IB	0	No	34	Single	Headache, tinnitus,	cerebrum	Surg+CT	NA
Peeples 1976	2/644	0.31%	NA	NA	NA	NA	NA	NA	NA	NA	NA	NA
van Nagell 1979	4/526	0.76%	NA	NA	NA	NA	NA	NA	NA	NA	NA	NA
Lefkowitz 1983	3		SCC (moderately)	IIIB	7	Yes(lung)	29	Multiple	Seizure, hemiparesis, paresthesia	cerebrum	WBRT	NA
			SCC (poorly)	NA	12	No	31	Single	Headache, diplopia, mandibular pain, facial numbness	cerebrum	Surg+WBRT+CT	NA
			ASC (moderately)	NA	39	Yes (bone, lung, vulva)	68	Single	confusion, speech disturbances, right hemiparesis	cerebrum	WBRT	NA
Friedman 1983	1		NA	IB	0.25	No	NA	NA	NA	NA	NA	NA
Gaze 1989	1		SCC (poorly)	IIIB	60	Yes(pelvis)	32	Single	NA	cerebrum	Surg+WBRT	18
Saphner 1989	6/1,219	0.49%	NA	NA		NA	NA	NA	NA	NA	NA	NA
Kumar 1992	2		AC	IIIB	48	No	48	Multiple	Headache, hemiparesis, facial palsy	cerebrum	WBRT+CT	2
			SCC	IIB	50	Yes (cervical LN)	50	Single	Headache, impaired memory	cerebrum	Surg+WBRT	7
Fagundes 1992	18/1,211	1.49%	NA	I (4), II (3) III (5), IV (6)	NA	NA	NA	NA	NA	NA	NA	NA
Cormio 1996	14/1,184	1.18%	NA	NA	18	NA	52	Single (6) Multiple (8)	Motor weakness, headache, seizures, dizziness, visual disturbance	Cerebrum (10) cerebellum (2) both (2)	Steroids (11) RT (3)	total 4 (1-21) RT 2,6,21
Robinson 1997	1		SCC (poorly)	IIIB	24	No	68	Single	Dizziness, headache, ataxia	cerebellum	Surg+WBRT	72
Salpietro 1998	1		SCC	NA	24	Yes (lung, hilar LN)	44	Single	NA	cerebrum	Surg	3
Senapati 1998	1		SCC (poorly)	IB	NA	Yes(lung)	45	Single	Headache, vomiting, hemiparesis	cerebrum	Surg+WBRT	NA

Ikeda 1998	8	SCC (poorly) (4)<br>SCC (moderately) (1)<br>AC (poorly) (2),<br>UD (1)	IB1(2),<br>IB2(1)<br>IIB (4)<br>IIIB (1)	24.6<br>(6.1-61.8)	Yes (8)<br>(lung (2), bone (3),<br>skin (1), LN (3))	56.5<br>(36-73)	Single (4)<br>Multiple (4)	Hemiparesis, headache,<br>vomiting, seizure	cerebrum	Surg+WBRT (3)<br>WBRT (5)	total 4.1<br>(1.8-22.6)<br>Surg+WBRT<br>7.5(4.1,7.5,10.3)
Ziainia 1999	1	SCC (poorly)	IIB	4	Yes (para-aortic LNs)	38	Single	Hemiballism	cerebrum	WBRT	4
Cormio 1999	1	SCC	IB	29	Yes (pelvic wall, lung,<br>liver)	51	Single	Headache, confusion, dizziness	cerebrum	Surg+CT	10
Cormio 2000	1	NA	NA	45	Yes(lung)	42	Single	NA	cerebrum	SRS	30
Mahmoud-Ahmed<br>2001	6	SCC (3)<br>ASC (2)<br>AC (1)	IB (1)<br>IIB (2)<br>IIIB (2)<br>IVB (1)	7.25<br>(0-18.5)	Yes (5)<br>(LN (2), lung (3), ribs<br>(3))	42<br>(39-69)	Single (2)<br>Multiple (4)	Headache	cerebrum (4)<br>both (2)	WBRT (3)<br>Surg+WBRT (1)<br>SRS (1)<br>SRS+WBRT (1)	total 7.75<br>(0.5-22.5)<br>Surg+WBRT<br>10.5<br>SRS 7, 22.5
Omari-Alaoui 2003	2	SCC (undiff)	IB	8	Yes (liver, lung, bone)	48	Single	Headache, vomiting, cerebellar<br>signs	cerebellum	Surg+WBRT	8
		SCC (poorly)	IIB	9	No	67	Multiple	Increased ICP, cerebellar signs	cerebellum	WBRT	2
Tajran 2003	1	AC	IB	96	Yes (Vagina, lung)	59	Single	Headache	cerebrum	WBRT	4.5
Salvati 2004	1	SCC (poorly)	NA	54	NA	48	Single	Arm focal seizure	cerebrum	Surg+WBRT+CT	11
Amita 2005	1	SCC (poorly)	IIA	46	No	54	Single	Decreased visual fields, ataxia<br>vomiting, headache	cerebrum	Surg+WBRT	6
Nagar 2005	1	AC (moderately)	IIA	9	Yes (omentum)	72	Multiple	Headache, hemiparesis	both	WBRT+ steroids	0.5
Cordeiro 2006	3	AC	IIB	-0.25	No	60	Single	None	cerebrum	Surg	NA
		SCC (poorly)	NA	NA	Yes (lung)	31	Single	Headache, hemianopsia	cerebrum	Surg+WBRT	60
		AC (poorly)	NA	24	Yes (abdomen)	31	Single	Headache, drowsiness,<br>visual deficits, diplopia, ataxia	cerebellum	WBRT	1
Gaussmann 2006	1	SCC (poorly)	I	36	Yes (lung, pleura, bone,<br>skin, mediastinal LNs)	36	Single	Gait disturbances	cerebrum	Surg+WBRT	120
Portera 2006	1	SCC	NA	156	Yes<br>(retroperitoneal,	43	Single	NA	cerebrum	WBRT+CT+ SRS	2.25

						paratracheal<br>cervical LN)						
Chura 2007	12	0.77%	SCC (8)<br>AC (4)	IB1 (5)<br>IB2 (1)<br>IIB (3), IIIA<br>IIIB, IVB	17.5<br>(1.1-96.1)	Yes (11) No (1)<br>(chest (8), pelvis (7)<br>abdomen (6), bone (4))	44.5<br>(31-58)	Single (4)<br>Multiple (6)<br>NA (2)	Headache, confusion,<br>seizures, paralysis	cerebrum	WBRT+steroids (8)<br>Surg+WBRT (1)<br>Steroids (3)	2.3<br>(0.3-7.9)
Agrawal 2007	1		SCC (moderately)	IIB	36	No	49	Multiple	Hemiparesis, headache, vomiting	cerebrum	Surg	NA
Brown 2007	1		ASC (poorly)	IB2	0.5	No	60	Single	Dysmetria, hemianopsia,<br>confusion	cerebrum	SRS+CT	5
Ogawa 2008*	7/1,716	0.40%	NA	NA	NA	NA	NA	NA	NA	NA	NA	2.8<br>(0.7-28.4)
Growdon 2008*	3		NA	NA	NA	NA	NA	NA	NA	NA	NA	3.5<br>(1-7.5)
Rades 2009*	9		NA	NA	NA	NA	NA	NA	NA	NA	WBRT	NA
Peters 2010	1		SCC	IIIB	NA	Yes (lung)	38	Single	Seizures, slurred speech<br>expressive aphasia, weakness	cerebrum	Surg	NA
Ding 2010	1		SCC (moderately)	IB2	6	Yes (spinal cord)	39	Single	Headache	cerebrum	Surg+WBRT	NA
Park 2010	1		SCC (poorly)	IB2	30	Yes (left SCLN)	48	Multiple	Headache, dizziness	both	WBRT+steroids	6
Menendez 2012*	2		NA	NA	NA	NA	42	Single	NA	NA	Surg+SRS (1)<br>SRS+CT (1)	5
Setoodeh 2012	2		SCC (poorly)	IVB	0	Yes (liver, lung)	53	Single	Aphasia, hemiparesis	cerebrum	Surg	Died<br>postoperatively
			SCC (poorly)	IVB	0	Yes (lung, bone)	43	Multiple	Headache	cerebrum	Surg+WBRT+CT	NA
Marongiu 2012	2		SCC (poorly)	IIIB	54	No	48	Single	Focal seizure	cerebrum	WBRT+CT	11
			SCNEC	IIB	18	No	34	Single	None	cerebrum	Surg	11
Lan-Fang 2012	4		SCNEC	NA	NA	NA	NA	NA	NA	NA	NA	NA
Yuan 2012*	6		NA	NA	NA	NA	NA	NA	NA	NA	NA	NA
Azimirad 2013	1		SCC (poorly)	IIB	24	Yes<br>(bladder, ureter	49	Single	Altered mental status	cerebrum	No	few days

						vaginal stump<br>rectum, colon<br>abdominal wall)			resting tremor<br>rigidity, bradykinesia			
Chung 2013	13		SCC (7), Small (3)<br>AC (1), NA (2)	IB (4), IIA<br>(3)<br>IIB (2)<br>IIIA (1)<br>NA (3)	38<br>(8.7-127.2)	Yes (11), No (2)<br>(Lung (8), bone (2)<br>abdomen (2), LN (1))	50<br>(31-79)	Single (4)<br>Multiple (9)	Headache, weakness,<br>dizziness, seizures<br>disturbed consciousness	both	SRS (7)<br>SRS+WBRT (6)	4.6<br>(1.9-15.9)
Hwang 2013	11/2,458	0.45%	SCC (5), AC (3),<br>ASC (1), Small (2)	IB (1), IIB<br>(2)<br>IIIA (1)<br>IIIB (2)<br>IVB (3)<br>NA (2)	15.4<br>(3.4-83.3)	Yes (11)<br>(lung (8), liver (4)<br>bone (2), SCLN (4)<br>mediastinal LN (2),<br>para-aortic LN (1))	50<br>(33-75)	Single (2)<br>Multiple (8)<br>NA (1)	Headache, left hemipareesia<br>aphasia, seizure, confusion<br>RUE weakness	cerebrum (5)<br>cerebellum (6)	CT (1), WBRT (6)<br>SRS+CT (1)<br>WBRT+CT (1)<br>Surg+WBRT (1)	5.9<br>(0.7-19)
Nasu 2013*	42		SCC (27)<br>AC (6)<br>ASC (2)<br>NEC (1)<br>Small (4)<br>NA (1)	I (14)<br>II (12)<br>III (5)<br>IV (10)<br>NA (1)	36<br>(0-386)	Yes (35), No (7)	53<br>(32-87)	Single (14)<br>Multiple (28)	NA	NA	Surg (2)<br>Surg+RT (5)<br>Surg+CT (1)<br>Surg+RT+CT (2)<br>RT (21)<br>WBRT+CT (3)<br>NA (6)	5
Vitorino-Araujo<br>2013	1		SCC	IIIB	NA	Yes (scalp, skull)	55	Single	NA	cerebrum	Surg	Alive
Shepard 2014*	1		NA	III	60	NA	40	Multiple	Nausea, vomiting	NA	WBRT	16
Tenjarla 2014	1		SCC	IV	0	Yes (lung. liver)	45	Multiple	Cranial nerve palsy, gait ataxia	cerebellum	WBRT+ steroids	1
Branch 2014	1		ASC (poorly)	III	72	Yes (lung, cervix)	46	Single	Seizure	cerebrum	Surg+WBRT	Alive
Erdis 2014	1		SCC	IV	NA	NA	67	Single	Headache	cerebrum	WBRT	NA
Kim 2015*	10		NA	I (1), II (1)<br>III (2), IV (6)	33.1<br>(0-84.3)	NA	43<br>(22-70)	Single (2)<br>Multiple (8)	NA	cerebrum (2)<br>cerebellum (8)	Surg (3), RT (10)<br>CT (7)	8.4<br>(6.6-10.1)

Pyeon 2015	1	SCC (poorly)	IIA2	8	No	44	Single	Headache, dizziness, ataxia	cerebrum	WBRT	7
Sato 2015	1	SCC (poorly)	IIB	0<br>4	Yes (cervix, liver, lungs, mediastinal LNs)	50	Multiple	Headache, vertigo, amnesia, vomiting<br>left hemiparesis, left facial paresis	both	Surg+WBRT+CT<br>SRS	7
Gressel 2015*	6	AC (1), SCC (3)<br>ASC (2)	I (1)<br>II (1)<br>IV (3)<br>NA (1)	42.5<br>(1-116)	Yes (6)<br>(lung (4), Bone (3)<br>liver (2), H&N (1))	52<br>(47-77)	Single (1)<br>Multiple (5)	NA	NA	WBRT (4)<br>Surg+WBRT (1)	3(1-22)<br>WBRT 6.5<br>(2-22)<br>Surg+WBRT<br>2.0
Walter 2015*	3	NA	NA	NA	Yes (3)	NA	NA	Headache, Ataxia<br>Altered mental status, seizures, stroke	NA	WBRT (1) Surg (2)	NA
Cacho-Diaz 2016	27	SCC (11), AC (7), ASC (2), Small (7)	I (5), IIB (17)<br>III (1), IV (3)	46<br>(3-248)	Yes (16) (lung (16)),<br>No (11)	50 ± 11<br>(33-70)	Single (9)<br>Multiple (14)	Headache, altered mental status, weakness, ataxia, nausea/vomiting, visual loss, cranial nerve palsy, language alteration, none, seizures	NA		8.2<br>(1-34)
Gupta 2016	1	ASC (poorly)	NA	0	Yes<br>(uterus, vagina, bladder)	52	Single	facial twitching, droop, thumb paresthesia	cerebrum	Surg+WBRT+CT	6
Shin 2016*	4	SCC (2), Small (2)	NA	NA	NA	NA	NA	NA	NA	SRS	3.5
Gilani 2016*	4	SCC (2), Small (2)	II (1), III (3)	25	NA	NA	NA	NA	NA	NA	3
Divine 2016*	19	NA	NA	NA	NA	NA	NA	NA	NA	NA	23
Dziggel 2016*	8	NA	NA	NA	NA	NA	NA	NA	NA	NA	NA
Keller 2016*	6	NA	NA	NA	NA	NA	NA	NA	NA	SRS	NA
Matsunaga 2016*	13	NA	NA	NA	NA	NA	NA	NA	NA	SRS	NA
Kim 2017*	2	NA	NA	99	NA	38	Single	NA	Cerebrum	Surg+WBRT	42
Johnston 2017*	2	NA	NA	NA	NA	NA	NA	NA	NA	SRS	5
		NA	NA	75	NA	43	Multiple	NA	Cerebrum	SRS	6

Takeshita 2017*	18/1,046	1.70%	SCC (8), AC (4),<br>NEC (4), CS (1)	I (2), II (6),<br>III (4), IV (6)	NA	Yes (16)	53	Single (8)<br>Multiple (10)	NA	NA	BSC (8), RT (7)<br>Surg+RT (2)<br>RT+CT (1)	2.75<br>(0.25-37.5)
Hayashi 2017*	33		SCC (19), AC (8),<br>SCC (3)	NA	33<br>(-11-114)	Yes (23), No (9)	58<br>(33-80)	Single (8)<br>Multiple (25)	NA	cerebrum (14)<br>cerebellum(15)	NA	NA
Fetcko 2017	1		SCC	IIIB	24	NA	75	Single	decreased coordination and<br>balance	cerebrum	Surg+SRS	6
Rades 2017*	8		NA	NA	NA	NA	NA	NA	NA	NA	NA	NA
Rades 2018*	1		NA	NA	NA	NA	NA	NA	NA	NA	NA	NA
Janssen 2018*	8		NA	NA	NA	NA	NA	NA	NA	NA	WBRT	NA
Gigliotti 2018*	2		NA	NA	NA	NA	NA	NA	NA	NA	SRS	NA
Bi 2019	2/1,800	0.10%	NEC	IIB	26	Yes	46	Single	Headache, vomiting, hemianopia	cerebrum	Surg+CT	9
Zhang 2019*	5		SCC (4), Small (1)	I (1), II (1)<br>III (2), IV (1)	NA	NA	52.8	Single (2)<br>Multiple (3)	Headache, altered mental status,<br>seizures, ataxia, syncope	cerebrum (3)<br>cerebellum (2)	NA	NA
Sadik 2019*	4		NA	NA	NA	NA	NA	NA	NA	NA	NA	NA
Kim 2019	19	0.68%	SCC (10), Small (4),<br>AC (3), ASC (1),<br>CS(1)	I (3), II(10),<br>III(4), IV(1)	19<br>(9-166)	No(3), Yes (16)	54.1<br>(31-75)	Single (11)<br>Multiple (8)	NA	NA	Surg+RT+CT(6)<br>RT(4), RT+CT(5)<br>CT(2), BSC(2)	6<br>(0-73)
Nasioudis 2020*	211/57,160	0.40%	NA	NA	NA	Yes (58), No (153)	54	NA	NA	NA	None (153)<br>WBRT (52)<br>SRS (<10)	4.99
Gardner 2020*	12/541	2.20%	NA	IV	NA	NA	NA	NA	NA	NA	NA	NA
Rades 2020*	2		NA	NA	NA	NA	NA	NA	NA	NA	NA	NA

AC: adenocarcinoma, ASC: adenosquamous carcinoma, BMs: Brain metastases, CT: chemotherapy, Cx: cervical cancer, LN: Lymph nodes, NA: Not applicable, SCC: Squamous cell carcinoma, Small: Small cell carcinoma, SRS: Stereotactic radiosurgery, Surg: Surgery, UD: Undifferentiated carcinoma, WBRT: Whole-brain radiotherapy

*: These reports include all gynecologic malignancies. We extracted information of only cervical cancer.

Supplementary table2. Clinicopathological features, treatments and outcome data of patients with brain parenchyma metastases from endometrial carcinoma documented in the literature.

Author	BMs(N)	Incidence	Histotype (N)	Grade (N)	FIGO stage (N)	Interval EC to BM (mo)	Other metastases	Median age (yrs)	Single BM/ multiple BMs (N)	Most common symptoms	Site of BM	Treatments	Median survival (months)
Salibi 1972	1		AC	NA	NA	6	No	63	Single	Seizure	cerebrum	Surg	18
Nakano 1975	1		AC	NA	NA	26	Yes	77	Single	Headache	cerebrum	Surg+RT	4
Hacker 1980	1		AC	NA	NA	216	No	64	NA	Diplopia	NA	Surg	0.8
Turner 1982	1		AC	G3	NA	NA	No	83	Single	NA	cerebrum	Surg	1
Aalders 1984	11/3,393	0.30%	NA	NA	NA	NA	Yes (3)<br>No (8)	NA	NA	NA	NA	NA	NA
Ritchie 1985	1		AC	NA	IIIC	NA	Yes	61	Multiple	NA	cerebrum	NA	4
Savage 1987	1		AC	G1	I	0	Yes	70	Single	NA	cerebrum	RT	14
Sawada 1990	1		AC	G3	IIIC	1.5	No	43	Single	Headache, diplopia	cerebrum	Surg+RT	84
Brezinka 1990	1		AC	G1	I	3	Yes	59	Single	Motor weakness	cerebrum	Surg	0.75
Kottke-Marchant 1991	3		AC	G3	IIIC	-3	Yes	59	Single	Seizure	cerebrum	Surg+RT	37
			AC	G3	IIIA	0	No	43	Multiple	Vision changes, headache	both	Surg	1
			AC	G3	IIIA	-2.5	No	46	Single	Seizure	cerebrum	Surg+RT	9
Thomas 1992*	1		AC	NA	I	24	No	51	Single	NA	cerebrum	Surg+RT	84
De Porre 1992	11/2,293	0.48%	ASC (1)<br>NA (10)	G3	NA	8 (1)<br>NA (10)	No (1)<br>NA (10)	67	Single (1)<br>NA (10)	Vision change	cerebrum (1)<br>NA (10)	Surg (1) NA (10)	14, NA (10)
Wronski 1993	2		AC	NA	NA	22	Yes	70	Multiple	Confusion, nausea, vomiting	cerebellum	Surg+RT	5.5
			AC	NA	NA	88	Yes	60	Multiple	Confusion, headache	both	RT	2
Ruelle 1994	2		AC	G3	NA	14	Yes	64	Single	Headache, gait disturbance	cerebellum	Surg+RT	9
			AC	G3	I	-0.25	No	63	Single	Headache	cerebrum	Surg+RT	24
De Witte* 1996			AS	G3	IIIC	NA	No	40	Single	NA	cerebrum	Surg+RT	NA

Cormio 1996	10/1,069	0.90%	AC (9)<br>ASC (1)	G3 (6)<br>NA (4)	IB (3) IC (2)<br>IIIA (2)<br>IIIC (1)<br>IV (2)	26<br>(3-81)	Yes (4)<br>No (6)	59<br>(47-71)	Single (6)<br>Multiple (4)	Motor weakness	Cerebrum (7)<br>cerebellum (1)<br>both (2)	Surg (1)<br>Surg+RT (2)<br>RT (1) Steroids (6)	1 (1-83)<br>Surg (3)<br>Surg+RT (28,83)<br>RT (3)<br>streoids (1,2,1,1,1,1)
De Witte 1996	2		ASC	NA	NA	24	No	67	Single	NA	cerebrum	Surg+RT	60
Salvati 1998	2		AC	G3	IA	10	No	48	Single	NA	cerebrum	Surg+RT+CT	20
			AC	G3	IA	26	No	54	Single	NA	cerebrum	Surg+RT+CT	74
Martinez-Manas 1998	1		AC	NA	IIB	18	No	76	Single	Vision changes	NA	Surg	8
Ogawa 1999	2		AC	G2	IIB	36	Yes	43	Multiple	NA	cerebrum	RT	5
			AC	G3	IIB	18	Yes	64	Multiple	NA	both	RT	3
Crispino 2000	1		AC	G3	IC	12	No	57	Single	NA	cerebellum	Surg+RT	3
Petru 2001	2		AC	G3	IV	NA	No	59	Single	Hemiparesis	cerebrum	SRS	171
			SC	G3	IIIC	NA	No	60	Single	Headache	cerebellum	SRS	17
Mahmoud-Ahmed 2002*	10/1,391	0.70%	AC (7)<br>ASC (3)	NA	IIA (1)<br>IIIA (1)<br>IIIB (2)<br>IIIC (2) IV (4)	8<br>(0.25-79)<br>NA (2)	Yes (7)<br>No (3)	51<br>(48-80)	Single (3)<br>Multiple (7)	NA	cerebrum (8)<br>cerebellum (1)<br>both (1)	SRS+RT (1) RT (4)<br>Surg (2)<br>Surg+RT (2)<br>Surg+SRS+RT (1)	3.25(0.25-15.5)<br>SRS+RT(1)<br>RT(0.25,2.25,2.5,6)<br>Surg+RT(15,15.5)<br>Surg+SRS+RT(11.5)
Sewak 2002	1		AC	G3	IB	48	Yes	63	Single	Dizziness, nausea	cerebellum	Surg+RT	
Shiohara 2003	1		AC	G3	IIIA	0	No	48	Single	Headache, vision changes	cerebrum	Surg+SRS+CT	6.5
Elliot 2004	1		AC	G3	IIB	2	No	51	Single	NA	cerebrum	Surg+RT+CT	38
Salvati 2004	2		AC	G2	IA	48	No	62	Single	Hemiparesis	cerebrum	Surg+RT	30
			AC	G3	IIIC	-0.5	Yes	51	Single	Hemiparesis	cerebrum	Surg+RT+CT	9
Gien 2004	8/1,295	0.60%	AC (7)<br>ASC (1)	G2 (3)<br>G3 (5)	IIB (2)<br>IIIC (4)<br>IVB (2)	8.5<br>(0-40)	Yes (6)<br>No (2)	67.5<br>(48-82)	Single (4)<br>Multiple (4)	Motor dysfunction	cerebrum (4)<br>cerebellum (2)<br>both (2)	RT (6) CT+RT (1)<br>steroids (1)	34

N'Kanza 2005	1		NA	NA	NA	NA	Yes	61	NA	Dizziness, weakness	NA	Surg+WBRT	3.5(0.25-7)<br>RT(0.5,1,2,5,5,7,7)<br>stroid(0.25)
Llaneza-Coto 2006	1		AC	G3	IIA	0	No	43	Single	NA	cerebrum	Surg	3
Lee 2006	1		AC	G3	IB	96	No	54	Multiple	Seizures	cerebrum	WBRT	1
Orru 2007	3/348	0.90%	AC	G3	IIIC	17	No	61	Multiple	NA	cerebrum	Surg+RT	0.25
			AC	G3	IIIB	10	No	49	Single	NA	cerebrum	Surg+RT	64
			AC	G3	IIIA	6	No	60	Single	NA	cerebrum	RT	16
Chura 2007	20/2,063	0.97%	AC (12)<br>ASC (2)<br>CS (3)<br>UD (3)	G1 (3)<br>G2 (6)<br>G3 (11)	IA (1) IB (2)<br>IIIA (4)<br>IIIC (4)<br>IVB (9)	11.5<br>(0.6-73.6)	Yes (16)<br>No (4)	64<br>(49-78)	Single (8),<br>Multiple (12)	NA	cerebrum (19)<br>cerebellum (6)	RT (7), RT+CT (4),<br>Surg+RT (1),<br>RT+SRS (1),<br>Surg+RT+CT (3),<br>No (4)	4
Sohaib 2007	1/86	1.16%	AC	NA	NA	NA	No	NA	Single	NA	NA	NA	2(0.1-39.2)<br>No(0.1,0.2,1.0,7.6)<br>RT,RT+CT(0.3-10.9)
Monaco 2008	6		NA	NA	NA	NA	NA	60.4	NA	NA	NA	SRS	NA
Ramirez 2008	1		AC	G3	IIB	12	No	61	Multiple	NA	cerebellum	RT	5(0.2-25)
Farooq 2008	1		AC	G1	IVB	-3	Yes	63	Single	NA	cerebrum	Surg	17
Al-Mujaini 2008	1		AC	NA	NA	84	Yes	69	Multiple	NA	cerebrum	NA	NA
Growdon 2008*	12		NA	G1 (1)<br>G2 (2)<br>G3 (8)	I (2) II (3)<br>III (4) IV (3)	NA	NA	NA	NA	NA	NA	NA	NA
Ogawa 2008*	4/556	0.70%	NA	NA	NA	NA	NA	NA	NA	NA	NA	NA	4
Rades 2009*	11		NA	NA	NA	NA	NA	NA	NA	NA	NA	WBRT	4.3(3.1-4.9)
Forster 2011	1		AC	NA	I	108	Yes	58	Multiple	NA	cerebrum	Olaparib	NA
Menendez 2012*	5		NA	NA	NA	NA	NA	66	NA	NA		SRS (4),<br>WBRT+SRS (1)<br>CT (3)	18

Yuan 2012*	5	NA	NA	NA	NA	NA	NA	NA	NA	NA		13
Nasu 2013*	39	AC (29)<br>ASC (1)<br>CS (4)<br>LS (4)<br>UD (1)	NA	I (10) II (2)<br>III (12) IV<br>(15)	25.2±<br>32.2<br>(0-156)	Yes (30)<br>No (9)	60.9±9.4<br>(39.8-77.9)	Single (17)<br>Multiple (22)	NA	NA	Surg (1)<br>Surg+RT (7)<br>Surg+CT (1)<br>RT (18)<br>RT+CT (2), CT (3)<br>No (7)	6.2
Gulsen and Terzi 2013	1	AC	G3	III	27	No	71	Multiple	NA	cerebellum	Surg+WBRT+CT	9
Colmenar Romero 2013	1	AC	G3	I	0	No	76	Single	NA	cerebrum	Surg+WBRT	2.5
Yoshida 2013*	1	AC	G1	I	NA	No	NA	Single	NA	NA	Surg+WBRT	48
Nassir 2014	1	AC	G2	II	24	No	72	Single	NA	cerebrum	Surg+WBRT	13
Shepard 2014*	6	NA	NA	NA	8	NA	77	Single	Seizure, altered mental status	cerebrum	SRS	16
				III	36	NA	32	Single	Syncope, blurry vision	cerebellum	SRS	8
				IV	21	NA	44	Multiple	Arm weakness	cerebrum	SRS	5
				IV	34	NA	52	Single	Dysarthria	cerebrum	WBRT	14
				I	29	NA	68	Multiple	Leg paresthesia	cerebrum	WBRT	6
				I	13	NA	79	Single	Altered mental status	cerebrum	Surg	4
Sierra 2015	1	AC	G3	III	12	No	55	Single	NA	cerebrum	Surg+WBRT+CT	8
Gressel 2015*	22	AC (17)<br>SCC (1)<br>ASC (1)<br>CS (2)<br>LS (1)	NA	I (1) II (2)<br>III (6) IV (12)<br>NA (1)	9.5	Yes (17)<br>No (5)	56.6	Single (8)<br>Multiple (14)	NA	NA	Surg (1),<br>RT (15)<br>Surg+RT (2)<br>No (4)	4<br>Surg 30<br>RT 4<br>Sug+RT 26<br>No 4
Kim 2015*	19	AC (17)<br>LS (2)	NA	II (3) III (8)<br>IV (8)	27.8<br>(0-53.4)	NA	58.2<br>(38.5-79.4)	Single (11)<br>Multiple (8)	NA	cerebrum (13)<br>cerebellum(6)	No (2), Surg (9)<br>RT (14), CT (9)	23.3<br>(17.8-28.8)
Kouhen 2015	1	AC	G3	I	24	No	62	Single	NA	cerebrum	WBRT+CT	30

Walter 2015*	13	NA	NA	NA	8.4	Yes (12)<br>No (1)	62	Single (6)<br>Multiple (7)	Headache, ataxia<br>altered mental status<br>dizzy, seizures<br>nausea, vomiting, weakness<br>stroke, vision changes	NA	WBRT (7), SRS (2)<br>SBRT (2)<br>Surg (1), BSC (1)	NA
Gilani 2016*	6	AC(3)<br>Sarcoma(3)	NA	I (2) III (2) NA<br>(2)	22<br>(3-45)	NA	62<br>(49-70)	NA	NA	NA	NA	7(1-27)
Uccella 2016	18	AC (15)<br>ASC (1)<br>UD (2)	NA	I (6)<br>IIIA (4)<br>IIIC (3)<br>IVB (5)	5<br>(1-57)	Yes (10)<br>No (8)	64	Single (9)<br>Multiple (9)	Headache, dysphasia,<br>weakness<br>confusion, seizures,<br>hemiparesis,<br>double vision, uncoordinated<br>movement	cerebrum (12)<br>cerebellum (2)<br>both (4)	SRS (1),<br>Surg+RT (8)<br>RT (5), RT+CT (1),<br>No (3)	6.5(0-118)<br>SRS (6)<br>Surg+RT (5-118)<br>RT, RT+CT<br>(1,2,3,5,17,28)<br>No (0,0.5,1)
Keller 2016*	10	NA	NA	NA	NA	NA	NA	NA	NA	NA	SRS	6
Shin 2016*	6	AC(5)<br>SCC (1)	NA	NA	NA	NA	NA	NA	NA	NA	NA	7.5
Divine 2016*	32	NA	NA	NA	NA	NA	NA	NA	NA	NA	NA	2
Dziggel 2016*	14	NA	NA	NA	NA	NA	NA	NA	NA	NA	NA	NA
Matsunaga 2016*	24	NA	NA	NA	NA	NA	NA	NA	NA	NA	SRS	NA
Kimyon 2017	1	UD (1)	G3	I	24	No	69	Single	NA	cerebrum	Surg+WBRT	17
Kim 2017*	4	NA	NA	NA	71	NA	55	Multiple	NA	cerebrum	SRS	6
		NA	NA	NA	20	NA	35	Multiple	NA	cerebrum	WBRT+SRS	28
		NA	NA	NA	60	NA	40	Multiple	NA	cerebrum	SRS	13
		NA	NA	NA	0	NA	54	Multiple	NA	cerebrum	SRS+WBRT	1
Hayashi 2017*	48	AC (34)<br>CS (5)<br>Others (7)<br>NA (2)	NA	NA	25<br>(-5-130)	Yes (36)<br>No (8)	60.5<br>(26-84)	Single (20)<br>Multiple (28)	NA	cerebrum (14)<br>cerebellum(15)	NA	8(5-15)

Takeshita 2017*	12/1,040	1.20%	AC (10)<br>CS (2)	NA	I (4), III (5), IV<br>(3)	NA	Yes (11)<br>No (1)	70<br>(54-78)	Multiple (5)	NA	NA	Surg+RT (3), RT (4),<br>RT+CT (1), No (4)	2.5(0.5-62)
Johnston 2017*	6		NA	NA	NA	NA	NA	NA	NA	NA	NA	NA	6(1-25)
Rades 2017*	14		NA	NA	NA	NA	NA	NA	NA	NA	NA	NA	NA
Kasper 2017	1		NA	NA	NA	NA	NA	NA	NA	NA	NA	SRS	NA
Janssen 2018*	13		NA	NA	NA	NA	NA	NA	NA	NA	NA	WBRT	NA
Rades 2018*	1		NA	NA	NA	NA	NA	NA	NA	NA	NA	NA	NA
Gigliotti 2018*	3		NA	NA	NA	NA	NA	NA	NA	NA	NA	SRS	NA
Moroney 2019	12		AC (11)<br>ASC (1)	G1 (1)<br>G2 (3)<br>G3 (8)	I (4)<br>II (1)<br>III (3)<br>IVB (4)	33<br>(7-199)	Yes (12)	52.5	Single (6)<br>Multiple (6)	Headache, focal weakness<br>Aphasia, seizures<br>vision changes, aphasia<br>hyponatremia, numbness<br>dizziness, confusion<br>balance issues, gait<br>disturbance<br>memory loss	cerebrum (11)<br>cerebellum (1)	Surg+RT (2),<br>Surg+RT+CT (2)<br>RT (4), RT+CT (2)<br>No (2)	3 (1-12)<br>Surg+RT,<br>Surg+RT+CT<br>(5,7,9,12)<br>RT, RT+CT<br>(1,2,3,3,7,10)<br>No (1,3)
Zhang 2019*	24		AC (17)<br>ASC (1)<br>CS (3)<br>LS (1)<br>sarcoma<br>(1)<br>NEC (1)	G1 (2)<br>G2 (2)<br>G3 (20)	I (3)<br>II (2)<br>III (12)<br>IV (7)	NA	NA	61.2	Single (8)<br>Multiple (16)	Headache, ataxia<br>weakness, altered mental<br>status<br>seizures, dizziness, syncope<br>numbness, aphasia	cerebrum<br>cerebellum (8)	NA	NA
Sadik 2019*	8		NA	NA	NA	NA	NA	NA	NA	NA	NA	SRS	NA
Nasioudis 2020*	498/243785	0.20%			NA	NA	Yes (165)<br>No (333)	61	NA	NA	NA	NA	4.34
Mao 2020	105/68,922	0.20%	AC (93)<br>CS (12)	G1 (6)<br>G2 (13)<br>G3 (45)	NA	NA	NA	NA	Single (41)<br>Multiple (64)	NA	NA	NA	5

Rades 2020*	3	NA	NA	NA	NA	NA	NA	NA	NA	NA	NA	NA

AC: adenocarcinoma, ASC: adenosquamous carcinoma, BMs: Brain metastases, CS: carcinosarcoma, CT: chemotherapy, EC: endometrial cancer, LN: Lymph nodes, LS: Leiomyosarcoma, NA: Not applicable, NEC: Neuroendocrine carcinoma, SCC: Squamous cell carcinoma, SRS: Stereotactic radiosurgery, Surg: Surgery, UD: Undifferentiated carcinoma, WBRT: Whole-brain radiotherapy

*: These reports include all gynecologic malignancies. We extracted information of only endometrial cancer.
